# Process visualization feedback in news translation: a controlled intervention study of human-AI interaction using multimodal process data

**DOI:** 10.3389/fpsyg.2026.1893830

**Published:** 2026-10-30

**Authors:** Xian Xian, Xun Guo, Wei Guo

**Affiliations:** 1 School of Foreign Languages, Hunan Institute of Engineering, Xiangtan, China; 2 School of Foreign Languages, Central South University, Changsha, China

**Keywords:** Inputlog, Krings' three-dimensional effort model, metacognitive awareness, news translation, process visualization feedback, strategic regulation

## Abstract

**Introduction:**

This study examines process visualization feedback in news translation.

**Methods:**

Framed within Krings' three-dimensional model, a pretest-posttest control group design was employed with 24 MTI students. The final analytic sample comprised 22 participants (11 experimental, 11 control). The experimental group (*n* = 11) received personalized process visualizations and structured reflection; the control group (*n* = 11) received conventional product-oriented feedback. Translation scores were available for both groups at T1 and T2, and complete process data (Inputlog, screen recording) were available for the experimental group at both time points.

**Results:**

Within the experimental group, within-subject comparisons showed changes in all three dimensions: in the temporal dimension, increased pause time proportion indicated heightened metacognitive engagement (*p* = 0.011); in the technical dimension, the meaning-level revision ratio increased significantly from 18.1% to 28.4% (*p* = 0.002), indicating a shift from surface-level correction to deeper substantive refinement; in the cognitive dimension, participants made more strategic use of external sources, with significant increases in search engine queries (*p* = 0.02) and knowledge-based searches (*p* = 0.01). Rich-points analysis of problem-triggered pauses revealed a shift from linguistic toward textual and extralinguistic problems (*p* = 0.038, *p* = 0.015). A mixed-design ANOVA revealed a significant Group × Time interaction (*p* = 0.038, η*p*^2^ = 0.198). Thematic analysis of reflective journals (*n* = 22) and interviews (*n* = 6) revealed two overarching themes: metacognitive awareness (54 references) of inefficient process patterns and competency gaps, and strategic regulation (60 references) in external source use and text-level restructuring.

**Discussion:**

These findings suggest that process visualization feedback was associated with enhanced metacognitive awareness and strategic regulation, and may support learners in becoming more reflective, strategic, and autonomous in human-AI collaborative news translation.

## 1 Introduction

When a breaking news story circulates globally, the version that reaches international audiences is rarely a verbatim translation of the original but a transformed product shaped by editors and translators who select, reorganize, and rewrite content under intense time pressure. News translation thus constitutes a critical domain where linguistic transfer intersects with ideological mediation, cultural adaptation, and rapid information communication (Bielsa and Bassnett, 2011; Károly, 2012). The primary objective of news translation requires practitioners to conform to target-language conventions and audience expectations while working within stringent deadlines (Bielsa and Bassnett, 2011; Schäffner, 2012). Within the Chinese educational context, news translation courses hold particular appeal for students, yet they simultaneously present substantial pedagogical challenges (Lim and Liao, 2015). Unlike other translation genres, news translation demands frequent adaptation of original texts to meet the needs and interests of specific readerships, resulting in operational principles that diverge significantly from traditional translation paradigms (Conway, 2015; van Doorslaer, 2010). The rapid advancement of neural machine translation (NMT) and large language models (LLMs) has fundamentally reconfigured this already complex landscape. These technologies affect students' engagement patterns and cognitive processing during translation tasks (Song and Song, 2023; Wang and Xue, 2024), while also reshaping educators' professional roles and instructional decision-making (Hamamra et al., 2025). In news translation specifically, output quality increasingly depends not only on linguistic and cultural competence but also on the ability to effectively manage interaction with AI tools throughout the production process (Yang et al., 2023; Jiménez-Crespo, 2025). The integration of AI into news workflows has intensified the need for translators who can strategically collaborate with machines rather than simply accepting machine-generated output. This human-AI collaborative competence has thus become a central objective of contemporary translator education (Ren and Wang, 2025; Wang et al., 2026).

Despite this growing imperative, students' translation processes, particularly how they collaborate with AI tools, remain largely invisible to both instructors and learners themselves. Traditional translation pedagogy has been predominantly product-oriented, focusing on the comparison of student work against instructor-provided model translations. Within conventional assessment frameworks, both instructors and students concentrate on final products rather than the processes that generate them, despite extensive evidence that process-level feedback is more powerful for deep learning (Nicol and Macfarlane-Dick, 2006; Hattie and Timperley, 2007; Hou and Li, 2013). Moreover, systematic analysis of students' human-AI collaborative competence is rarely conducted, limiting opportunities for targeted developmental feedback (Ren and Wang, 2025). This product-oriented focus becomes particularly problematic in the AI era, where effective collaboration with AI tools requires process-level strategic regulation, including appropriate time allocation, targeted revision behaviors, and strategic use of resources rather than mere product-level error correction (Krings, 2001; Mossop, 2014). Process visualization feedback, which converts behavioral traces into graphical representations, has been shown in L2 writing research to raise metacognitive awareness and foster self-regulation (Ranalli et al., 2018; Bowen et al., 2022; Vandermeulen et al., 2023). Translation process research has similarly employed keystroke logging, screen recording, and eye-tracking to analyze translators' cognitive processes through external behaviors (Feng, 2016; Li, 2017).

Nevertheless, a significant gap persists. While methods for collecting and analyzing multimodal process data have matured considerably, research on converting such data into targeted pedagogical interventions in news translation education remains limited (Sfeir et al., 2026). Furthermore, only a small number of studies on process-oriented feedback have combined such feedback with a controlled comparison group, which makes it difficult to determine how far observed improvements in product quality reflect the feedback intervention rather than task practice or temporal maturation (Vandermeulen et al., 2023). This limits the extent to which effects on learning outcomes can be attributed to the intervention itself. To address these gaps, the present study employs a controlled pretest-posttest intervention design in which the two levels of inference are kept separate: the between-group comparison tests the effect of the intervention on the quality of the translated product, while the process data are used to trace the mechanisms of change within the intervention group rather than to establish between-group effects. Specifically, this study seeks to answer the following research questions:

(1) Process-level changes: how do students' translation process behaviors, including temporal allocation, revision patterns, and resource consultation strategies, change after receiving process visualization feedback?
(2) Outcome-level effects: what is the impact of process visualization feedback on the quality of students' translated news texts?
(3) Perceptual and metacognitive effects: how do students perceive and engage with process visualization feedback, and how does such feedback influence their self-reported translation strategies and metacognitive awareness regarding human-AI collaboration?

## 2 Literature review

### 2.1 News translation in the AI era

News translation differs fundamentally from other translation genres. It involves substantial transformation of source texts, often producing target language content that diverges significantly from the original (Bielsa and Bassnett, 2011; Schäffner, 2012). The term “transediting” was introduced to describe this gray area between translating and editing, capturing how practitioners actively reorganize, condense, supplement, and rewrite content rather than simply producing target language equivalents (Stetting, 1989). However, this study adopts the term “news translation” to refer holistically to translation, editing, and rewriting practices, recognizing that all translation involves transformation (Schäffner, 2012).

The emergence of neural machine translation (NMT) and large language models (LLMs) has profoundly reconfigured the news translation landscape. Since the release of ChatGPT in late 2022 and DeepSeek in early 2025, machine translation post-editing (MTPE) is evolving toward AI-powered post-editing (AIPE), a mode characterized by enhanced interactivity and advanced intelligence (Wang and Liu, 2025). Translators increasingly work with AI-generated drafts rather than translating from scratch, spending less time on initial text production and more on evaluating, correcting, and refining AI output (Briva-Iglesias and O'Brien, 2024; Jiménez-Crespo, 2025; Wang and Liu, 2025). This transformation has elevated human-computer interaction (HCI) to a central concern in translation studies, as the translator-technology interface fundamentally shapes both processes and outcomes (O'Brien, 2012; Jiménez-Crespo, 2025). A human-centered AI approach emphasizes that translation competence should focus precisely on areas where machines exhibit limitations, such as creativity, narrativity, contextualization, and judgment, while positioning the human at the center of the cognitive system with ultimate control and autonomy (Briva-Iglesias and O'Brien, 2024; Jiménez-Crespo, 2025).

Research examining the quality implications of this integration has revealed both the potential and limitations of machine translation. Specific post-editing operations required for news translation include terminological correction, disambiguation, word order adjustment, sentence restructuring, and cultural and ideological adaptation (Feng and Li, 2016; Krüger, 2022). These findings indicate that post-editing news MT output demands not only linguistic competence but also considerable cultural sensitivity and editorial judgment, and thus poses particular challenges for novice translators (Cadwell et al., 2018; Yang et al., 2023). Consequently, cultivating effective human-AI collaboration competence has become a central objective of contemporary translator education (Mellinger, 2017; Ren and Wang, 2025).

Developing this competence, however, requires attention not only to students' evaluation of AI-generated output but also to how they engage with translation technologies. The availability of interactive LLMs does not automatically lead students to move beyond familiar lookup practices toward active prompting. Established habits with incumbent tools and perceived switching costs can generate inertia and weaken intentions to adopt new systems (Polites and Karahanna, 2012), while student translators often lack prompt-writing experience and may need repeated attempts before obtaining satisfactory output (Abdelhalim et al., 2025). Together, these factors may explain why students continued to rely on familiar MT lookup rather than engaging in sustained LLM dialogue.

### 2.2 Human-computer interaction in news translation

A three-dimensional effort model classifies post-editing effort into three interrelated dimensions: temporal effort (time required to complete a task), cognitive effort (the mental processing demands involved), and technical effort (the number and type of editing operations performed; Krings, 2001). This model has guided numerous empirical investigations into how translators interact with MT systems and provides the theoretical framework for the present study. The three indicators examined in this study, namely time allocation, revision behavior, and resource consultation, thus represent an operationalization of Krings' three-dimensional effort model within the specific pedagogical context of process visualization feedback.

Temporal effort is rooted in translation process research examining how translators distribute time across different phases of task completion. Professional translators and student translators differ markedly in how they allocate time across orientation, drafting, and revision phases (Jakobsen, 2002; Carl et al., 2016). In machine translation post-editing, temporal measures have been widely adopted to evaluate task difficulty and cognitive load, with evidence suggesting that time spent on individual segments correlates with both source text complexity and translator expertise (Koponen, 2016; Daems et al., 2017). More recently, researchers have argued that increased pause time may indicate heightened metacognitive engagement rather than inefficiency, as translators pause to plan, evaluate, and problem solve (Sfeir et al., 2026).

Technical effort connects to revision behavior and the nature of editing operations performed on translated texts. A comprehensive classification of revision strategies distinguishes between different types of edits and their functions, including surface level corrections (for example, spelling and punctuation) vs. deeper structural revisions (Mossop, 2014). In post-editing research, the number, type, and temporal distribution of edits serve as reliable indicators of both output quality and the degree of human engagement with machine generated text (Green et al., 2013; Daems et al., 2017). For news translation, revision patterns are particularly significant because the genre demands not only linguistic accuracy but also stylistic appropriateness and cultural adaptation (Feng and Li, 2016; Krüger, 2022). The ability to move beyond surface level error correction toward deeper structural and stylistic revisions distinguishes more effective from less effective news translators (Mossop, 2014; Vandermeulen et al., 2023).

Cognitive effort refers to the mental processing demands involved in translation and post-editing tasks. Although cognitive effort cannot be measured directly, it can be inferred from external behaviors such as source consultation, problem solving pauses, and resource use (Krings, 2001). In translation process research, information seeking behaviors, including dictionary lookups, search engine queries, and parallel text consultation, have been extensively analyzed as indicators of cognitive effort (Ranalli et al., 2018). The concept of rich points, moments in the translation process that reveal underlying difficulties or decision making, has been proposed to capture how translators navigate linguistic, textual, and extralinguistic challenges (Sfeir et al., 2026).

AI tool use represents a more recent research focus alongside the rapid development of NMT and LLMs. Investigations have examined how professional translators adopt and integrate machine translation into their workflows, revealing complex patterns of resistance and accommodation shaped by task characteristics, tool usability, and individual attitudes toward technology (Cadwell et al., 2018; Vieira, 2020). In educational contexts, research has examined how student translators interact with MT and what strategies they employ when post-editing (Yang et al., 2023; Jiménez-Crespo, 2025). Studies have shown that while post-editing reduces processing time and mental workload compared to human translation from scratch, novice translators often demonstrate inadequate proficiency in identifying and correcting MT errors (Mellinger, 2017; Yang et al., 2023).

While these dimensions of effort have demonstrated clear value for research purposes, their pedagogical implications in news translation have received considerably less attention. Educators have gradually recognized the need to integrate technology-related competences into training curricula (Mellinger, 2017; Hamamra et al., 2025), yet few studies have developed effective instructional approaches capable of making the human-computer interaction process itself transparent for students. Learners engaged in news translation often lack awareness of their own process patterns regarding time allocation, revision behaviors, and resource consultation strategies, and have limited opportunities for structured reflection on how they interact with AI tools and other resources throughout the task (Lindgren et al., 2009; Sfeir et al., 2026). This gap underscores the necessity of exploring instructional methods that can render the news translation process visible to learners.

### 2.3 Process visualization feedback

Feedback is commonly conceptualized as information provided to a learner regarding the extent to which a task or action has been carried out successfully (Sadler, 1989), and it has long been recognized as one of the most powerful influences on learning (Hattie and Timperley, 2007; Wisniewski et al., 2020). Feedback serves as a catalyst for the self-regulated learning cycle by providing external information that learners can use to evaluate the effectiveness of their strategies and adaptively adjust their approaches in subsequent tasks (Butler and Winne, 1995). Feedback directed at the process and self-regulation levels is considerably more powerful than task-level feedback when the aim is to promote deep processing and transferable strategic competence (Nicol and Macfarlane-Dick, 2006; Hattie and Timperley, 2007). Effective feedback must empower learners to monitor and regulate their own learning, rather than simply inform them of deficiencies in their products (Black and Wiliam, 1998; Nicol and Macfarlane-Dick, 2006).

Process visualization feedback has emerged as a promising pedagogical approach for rendering invisible composing and translating processes visible, analyzable, and actionable (Sullivan and Lindgren, 2006; Leijten and Van Waes, 2013). By transforming raw behavioral data into intuitive graphical representations, such feedback enables learners to examine their own operational patterns and identify areas for strategic refinement. Keystroke logging applications like Inputlog externalize the black box of the writing process, providing fine-grained information on production fluency, pausing behavior, revision patterns, and source consultation at different intervals across the writing process (Sullivan and Lindgren, 2006; Leijten and Van Waes, 2013). Recent developments within Inputlog have enabled the automatic generation of user-friendly process reports that translate raw logging data into accessible numerical summaries and graphical representations suitable for pedagogical use (Vandermeulen et al., 2020; Bowen et al., 2022).

Within second language writing research, scholars have demonstrated that data-driven depictions of composing processes, including pause patterns, revision trajectories, and resource consultation behaviors, can effectively raise metacognitive awareness and foster strategic self-regulation (Ranalli et al., 2018; Bowen et al., 2022; Vandermeulen et al., 2023). Keystroke logs and process graphs can make patterns of time allocation, pausing, and revision visible, offering a basis for reflection on writing processes and the development of learner autonomy (Xu and Xia, 2021). Real-time fluency-focused feedback based on keystroke logging has yielded positive effects, with students in intervention conditions producing more text and writing higher-quality texts than control groups (Speltz and Chukharev-Hudilainen, 2021). Providing students with personalized process reports yields meaningful improvements in text quality. Such visualization of the writing process allows learners to move beyond simple error correction toward more goal-oriented reading and writing strategies (Bowen et al., 2022; Vandermeulen et al., 2023). Process visualizations may support the development of metacognitive skills such as self-directed learning by making learners' own creative processes available for reflection and strategic planning (Adhikari, 2023). Self-regulated learning can be conceptualized through Winne and Hadwin's (1998) COPES framework, in which conditions shape operations, operations generate products, and comparisons between products and standards yield evaluations that may inform subsequent regulation. Process visualizations make behavioral traces available for such evaluation, although traces alone do not reveal learners' internal standards or reasoning (Winne, 2010). Internal feedback arises when learners compare their work with reference information, and such comparison is more likely to be productive when it is made explicit (Nicol, 2021). Process visualizations combined with structured reflection may therefore help learners compare recorded activity with task requirements and personal goals and formulate strategic adjustments for later tasks.

In the field of translation, process-oriented feedback has gained attention, though research remains less extensive than in L2 writing. A teaching experiment employing eye-tracking and keystroke logging enhanced MTI students' strategic competence through the playback and reflection of their own translation processes (Feng, 2016). Screen recordings can be used to reverse engineer translation products for purposes of process-oriented assessment, linking observable indicators such as information retrieval patterns and revision sequences to error triggers in the translation process. A product- and process-oriented analytical framework for examining pause placement patterns in translation keystroke data has demonstrated how such process indicators can reveal moments of translation difficulty and meaning construction in real time (Sfeir et al., 2026). Computer keystroke logging coupled with peer-based intervention has been shown to support student reflection and discussion around translation tasks while enhancing motivation and enthusiasm for translation (Lindgren et al., 2009).

Despite growing recognition that process visualization feedback can foster metacognitive awareness and strategic refinement in L2 writing and translation training, scant attention has been devoted to examining whether providing such process data back to learners in visualized form leads to tangible improvements in news translation specifically, particularly across the three dimensions of effort: temporal (time allocation and pausing behavior), technical (revision patterns), and cognitive (resource consultation and problem solving). Equally underexplored is how such feedback shapes learners' metacognitive monitoring and strategic regulation when working with AI and other resources in translation tasks. Controlled comparisons have also been uncommon in this line of work, which makes it difficult to separate the specific effects of process visualization feedback from general practice effects or temporal maturation (Vandermeulen et al., 2023). To address these gaps, the present study adopts a pretest-posttest control group design to examine whether process visualization feedback is associated with greater improvement in news translation quality, and draws on process data from the experimental group, operationalized along the three dimensions of Krings' (2001) model, to trace the behavioral changes observed in that group.

## 3 Methodology

### 3.1 Research design

This study employed a pretest-posttest control group design with matched assignments to examine the effects of process visualization feedback on the quality of the translated news product and on the translation process behaviors of the intervention group. The independent variable was feedback type: process visualization feedback derived from Inputlog and screen recording data (experimental group) vs. conventional product-oriented feedback (control group). The dependent variables comprised process-level indicators, including time allocation, AI tool usage, and revision patterns, and outcome-level indicators, including translation quality scores assessed by independent professional raters.

Both groups completed a pre-test translation task (T1), during which multimodal process data were collected from all participants. Following T1, participants were matched and assigned to either group based on their T1 translation quality scores. One participant in the experimental group was subsequently excluded due to corrupted process data, and to preserve the matched-pair structure, one control participant was also removed from the analysis to keep the two groups comparable in size, yielding final groups of 11 and 11. During the eight-week intervention period, the experimental group received personalized process visualizations and engaged in structured reflection, while the control group received conventional product-oriented feedback. At post-test (T2), both groups completed a parallel translation task. Process data were collected only from the experimental group to evaluate within-subject changes, while outcome data were collected from both groups to enable between-group comparison. The T2 process measures were designed to answer RQ1, that is, to document whether and how the feedback changed students' own translation behavior, for which the experimental group is the relevant population; the between-group test of the intervention effect rests on the outcome measure, which was obtained from both groups. As only the experimental group was tracked at T2, the RQ1 findings are interpreted as within-subject changes in the intervention group rather than as a between-group comparison.

### 3.2 Participants

A total of 24 Master of Translation and Interpreting (MTI) students enrolled in a selective news translation course at a 985 university in China participated in the study. All were first-year MTI students who had completed foundational translation courses prior to the study. The news translation course ran for one academic semester (16 weeks), and participation in the research component (8 weeks) was voluntary. Written informed consent was obtained from all participants following ethical guidelines for human subject research.

To form equivalent groups, participants were matched and assigned based on their T1 translation quality scores. After assignment, the Inputlog process data for one participant in the experimental group were found to be corrupted due to a technical failure. This participant was therefore excluded from the experimental group for intervention delivery and process analysis, and the matched counterpart in the control group was also removed from the analysis. The final experimental group consisted of 11 participants (8 females, 3 males) with complete T1 and T2 process data and translation scores. The control group therefore comprised 11 participants (9 females, 2 males) with complete T1 and T2 translation scores, giving a final sample of 22 participants. Table 1 presents demographic characteristics and baseline T1 scores for both groups.

**Table 1:** Demographic Information and baseline translation scores.

Characteristic	Experimental Group (*n* = 11)	Control Group (*n* = 11)
Gender (females/males)	8/3	9/2
T1 translation quality mean (SD)	90.36 (4.38)	90.45 (3.45)
T1 score range	80–96	85–95

T1 scores are reported on a 0–100 scale. An independent-samples *t*-test confirmed no significant difference in T1 scores between the two groups, *t* (20) = 0.05, *p* = 0.957.

### 3.3 Materials and instruments

#### 3.3.1 News translation tasks

Two parallel news translation tasks were designed for the pre-test (T1) and post-test (T2). Both were authentic news articles selected from reputable international news agencies to ensure ecological validity. The T1 task was a feature report titled “Chinese balloon soars across US; Blinken scraps Beijing trip” (*Associated Press*, February 4, 2023; 1,028 words), covering diplomatic tensions, military terminology, and ideological mediation. The T2 task was a feature report titled “China is all in on green tech. The U.S. and Europe fear unfair competition” (*The Washington Post*, March 29, 2024; 1,287 words), addressing economic terminology, trade policy discourse, and multiple stakeholder perspectives.

The comparability of the two source texts was established through a multi-dimensional assessment of readability and lexical properties (Table 2). Four standard readability indices (FRE, FKGL, ARI, SMOG) yielded highly similar results, with FKGL, ARI, and SMOG differing by less than one grade level and FRE by fewer than two points, indicating equivalent sentences and word-level complexity. Lexical measures further confirmed this alignment: type-token ratio, tokens per type, and lexical density showed near-identical values across texts, suggesting comparable lexical diversity and information density. Additional indicators of lexical variation and character-based complexity also exhibited negligible differences. While minor variation was observed in text length and sentence count, these did not translate into meaningful differences in overall complexity. Thus, the two texts were considered comparable at the level of surface linguistic and lexical features. Beyond these quantitative measures, both texts contained ideologically sensitive content requiring editorial judgment, a characteristic central to authentic news translation tasks (Bielsa and Bassnett, 2011; Schäffner, 2012). Each task was allocated 70 min, consistent with real-world news translation deadlines and the course's standard assessment format. The difficulty level was further informed by pilot testing with five MTI students who did not participate in the main study.

**Table 2:** Text comparability analysis.

Measurement	Indicators	Text 1	Text 2
General level	Number of characters	7,225	8,170
	Number of sentences	38	54
	Average sentence length	34.95	27.78
Lexical level	Character richness (TTR)	0.461	0.447
	Lexical variation (Verb)	0.131	0.131
	Number of characters occurring only once	371	387
Syntactical level	Mean length of prepositional phrases	2.099	2.469
	Mean number of non-phrase per simple clause	24.976	22.167
	Mean number of verb phrase per simple clause	3.5	3.111
	Mean number of simple clauses per sentence	0.929	0.852
Readability indices	FRE	39.78	41.64
	FKGL	13.94	13.57
	ARI	15.09	14.93
	SMOG	15.08	14.27

All measures were computed on the English source texts. “Number of characters” refers to English characters.

#### 3.3.2 Data collection tools

Inputlog (version 8.0) was installed on all laboratory computers to record participants' translation processes. The software captured every keystroke and mouse event. The pause threshold was set at 2 s, following established practice in writing process research (Speltz and Chukharev-Hudilainen, 2021). Key variables included action time (active keystrokes and mouse events), pause time (idle time exceeding 2 s), revision operations (deletions, insertions, substitutions), and source consultation events (task switching to browsers, dictionaries, or AI tools). EV Screen Recorder (version 4.2.3) captured full-screen video of participants' activities at 15 frames per second, with desktop audio disabled. Screen recordings were synchronized with Inputlog logs using system timestamps, enabling multimodal analysis of AI tool interaction (prompt input, copying/pasting MT output), dictionary lookups, and cross-referencing of online resources.

Participants in the experimental group completed structured reflective journals immediately after both T1 and T2. The T1 reflection prompted participants to rate task familiarity and difficulty on 1-to-5 Likert scales and to describe difficulties encountered and strategies used. The T2 reflection focused on changes in translation processes, AI tool use strategies, and metacognitive awareness. All 11 experimental group participants submitted both T1 and T2 journals, yielding 22 journals in total. From these, six students representing high-, mid-, and low-performing strata based on T1-to-T2 gain scores were selected for semi-structured interviews after T2. Journals and interviews served as qualitative data for triangulation.

#### 3.3.3 Intervention materials

Following T1, each experimental group participant received a personalized process visualization report generated from their Inputlog data using the Inputlog Data Analyzer and screen recording. Each report contained three components: (a) a temporal visualization representing the writing process. By receiving this visual feedback, students could compare their perceived process with the actual recorded behavior, identify overall personal strengths and weaknesses (e.g., excessive pausing, frequent source switching, or inefficient revision patterns), and ultimately use this awareness to self-regulate their future writing or translation strategies; (b) a behavioral visualization of external source consultation. This figure, by making normally invisible consultation patterns visible, enabled students to self-diagnose strengths and weaknesses in their information-seeking behavior and identify opportunities for more strategic information seeking; (c) an individual revision trajectory distinguishing surface-level edits (spelling, punctuation) from meaning-level revisions (syntactic restructuring, lexical substitution) and a revision intensity heatmap. These two figures combined allowed students to assess whether their revisions focused primarily on error correction or on deeper textual refinement and identify the proper timing of revision during news trans-editing process. A structured reflection log guided participants to identify three observations from their report, diagnose one or two areas for strategic improvement, and formulate specific action points for T2.

#### 3.3.4 Assessment tools

News translation prioritizes editorial adaptation, including selection, condensation, reorganization, and cultural reframing over equivalence-oriented transfer (Bielsa and Bassnett, 2011; Schäffner, 2012). Generic MT evaluation metrics like COMET or BLEURT, designed to measure similarity to reference translations, are ill-suited for this genre. Therefore, the study adopted a human rater approach, recruiting two senior news editors from *Huanqiu Net*, a major Chinese online news platform. Both editors had over 10 years of professional experience adapting international news reports for Chinese domestic audiences.

Both editors drew on their professional experience to form a single overall judgement for each translation, rather than scoring separate dimensions. Their judgement reflected, among other things: (a) factual accuracy and completeness, including the correct rendering of proper nouns, technical terms, and figures, and the absence of distortion or omission of source information; (b) conformity to Chinese news discourse conventions, covering headline and lead formulation, information hierarchy, attribution, register, and tone; (c) the quality of editorial adaptation, that is, whether the translator selected, condensed, reorganized, and rewrote the source rather than transferring it in a source-oriented manner; (d) acceptability to the target readership, covering naturalness, fluency, absence of translationese, and immediate comprehensibility for domestic readers; and (e) the handling of culturally or politically sensitive content, including whether the framing and mediation of such content was appropriate for a Chinese news platform. The 0–100 scale corresponded to the following levels of editorial readiness: 90–100, publishable with minimal editing; 80–89, publishable after light editing, with only minor problems in one or two criteria; 70–79, requiring moderate editing because of noticeable problems in accuracy, discourse organization, or acceptability; 60–69, requiring substantial rewriting before publication; and below 60, not suitable for publication. No dimension was scored separately; the aspects above were weighed together in a single holistic judgement, and each translation therefore received one score per rater.

A calibration phase preceded formal rating. Five translations were randomly selected from the T1 dataset and independently rated by both editors using a holistic 0-100 scale, assessing overall suitability for publication on a Chinese news platform. The intraclass correlation coefficient (ICC) for absolute agreement was 0.86, indicating good reliability. After resolving minor discrepancies through discussion, the editors independently rated the remaining T1 translations. Each participant's final T1 score was the mean of the two editors' ratings.

Eight weeks later, the same editors independently rated all 22 T2 translations (one participant was excluded from the experiment and one control participant was removed to equalize group sizes), remaining blind to group assignment, time point, and participant identity. Inter-rater reliability for the full set of 44 translations (22 participants × 2 time points) was ICC = 0.89 [95% CI (0.84, 0.93)], confirming high agreement. The final score for each translation at each time point was the arithmetic mean of the two ratings.

### 3.4 Procedures

The study was conducted over 8 weeks. In Week 1, all 24 participants completed the T1 translation task in a computer laboratory under controlled conditions, with Inputlog and screen recording active. Participants could access any digital resources they would normally use, including online dictionaries, MT tools (DeepL, Google Translate), and LLMs (ChatGPT). The task was allocated 70 min. Immediately after submission, participants completed the reflective journal.

Following T1, participants were matched and assigned to groups based on translation quality scores. During Weeks 2–7 (intervention period), each experimental group participant (*n* = 11) received an individual 30-min feedback session in Week 2, reviewing their process visualization report and using the structured reflection log to articulate action plans for T2. Participants received copies of their report and log for ongoing reference. In Weeks 3–7, they engaged in self-monitored practice through regular course assignments without structured feedback but were encouraged to implement their action plans. The control group (*n* = 11) received conventional product-oriented feedback, written instructor comments on their T1 translations focusing on accuracy, clarity, and editorial quality, following standard course practice. In Week 8, both groups completed the T2 translation task under identical conditions to T1. Inputlog and screen recording were shown only to the experimental group. Immediately after submission, the experimental group completed the post-task reflective journal. Semi-structured interviews were then conducted with six selected experimental group participants, audio-recorded, and transcribed verbatim.

### 3.5 Ethical considerations

This study was approved by the Ethics Committee of the authors' university. All participants provided written informed consent, acknowledging that Inputlog and screen recordings would capture all on-screen activities, that data would be anonymized, that participation was voluntary and could be withdrawn at any time without academic penalty, and that anonymized data might be shared for replication purposes. Real names were replaced with codes in all datasets and publications.

## 4 Results

The data analysis was structured into three complementary strands corresponding to the three research questions. The first strand addressed process-level changes (RQ1) and was organized according to Krings' (2001) three-dimensional model of post-editing effort. The second strand addressed outcome-level effects (RQ2) through mixed ANOVA comparing translation quality scores between groups and across time. The third strand addressed perceptual and metacognitive effects (RQ3) through thematic analysis of reflective journals and interview transcripts, triangulated with screen recording data.

### 4.1 Analysis of process-level changes (RQ1)

To examine how students' translation process behaviors changed from T1 to T2 in the intervention group, multimodal process data were analyzed across the three dimensions of Krings' model.

#### 4.1.1 Temporal effort

The first dimension of Krings' model, temporal effort, was operationalized as the time participants spent actively engaged in the translation task. A temporal visualization representing the translation process, generated by Inputlog's user report function, showed a graphic representation of the dynamics characterizing the student's writing process. A sample of this temporal visualization is presented in Figure 1 as part of the process visualization feedback.

**Figure 1:**
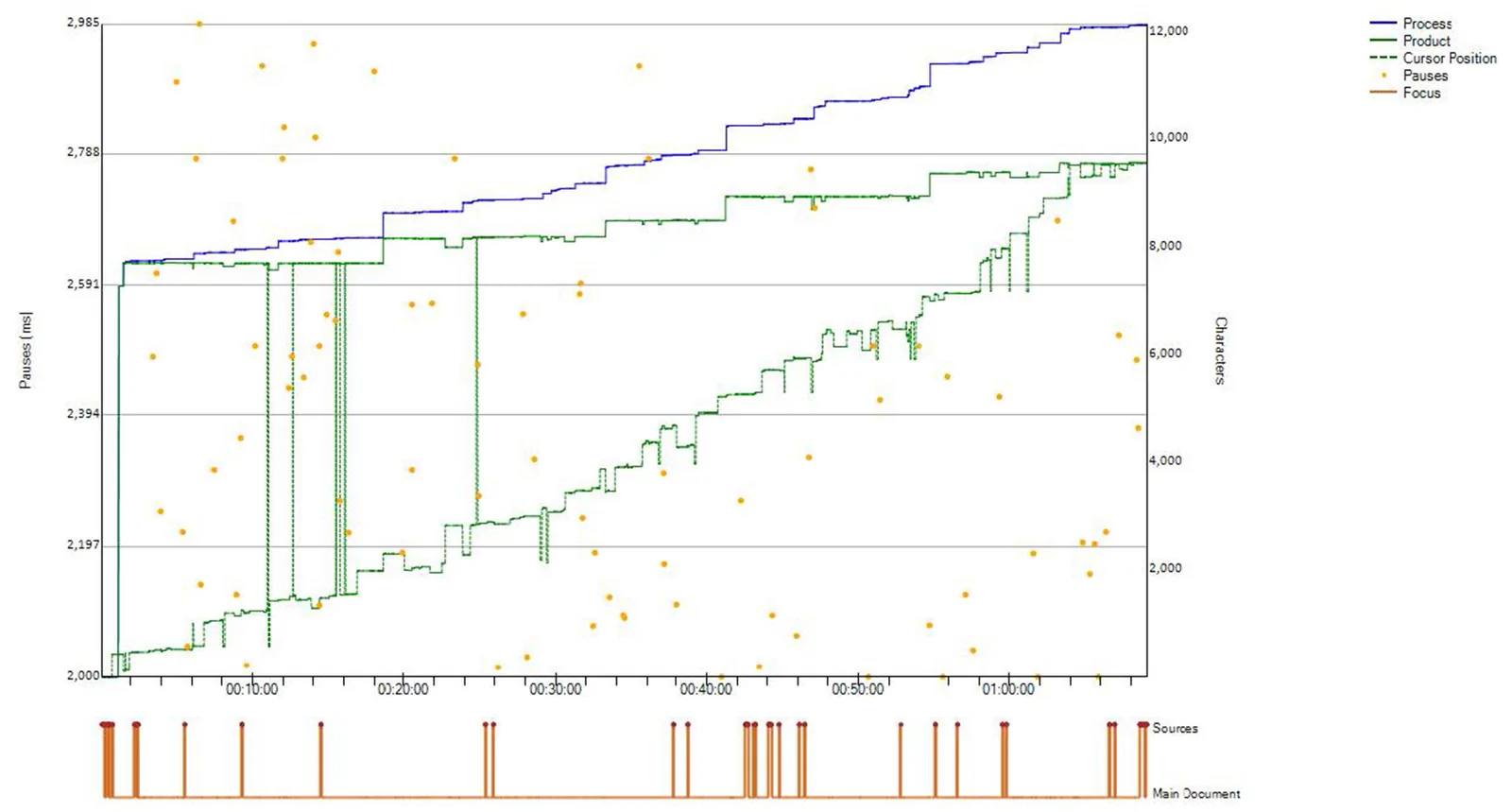
Temporal representation of the translation process. Shown for an example participant. The upper panel plots cumulative characters produced (green) and overall process progression (blue) against elapsed task time, with yellow markers indicating pauses; the lower panel shows the timing of source-consultation events (orange) and work on the main document.

The quantitative temporal indicators were measured using Inputlog's action time and pause time metrics. Action time was defined as the total duration of active keystrokes and mouse events excluding pauses. Pause time was defined as the total idle time exceeding the two second threshold, following established practice in writing process research (Speltz and Chukharev-Hudilainen, 2021). A pause time proportion was calculated as pause time divided by total process time. For each of the 11 experimental group participants, pause time and pause time proportion were extracted from the Inputlog General Log files for both T1 and T2. Paired-samples t-tests were conducted to compare T1 and T2 measures within the experimental group, with effect sizes reported as Cohen's *d*.

As shown in Table 3, the experimental group demonstrated a significant increase in the pause time proportion from T1 to T2. The proportion rose from 25.0% (SD = 12.0%) at T1 to 33.4% (SD = 14.5%) at T2, a mean increase of 8.4 percentage points (*p* = 0.011, Cohen's *d* = 0.61). These results indicate that participants spent proportionally more time pausing during the T2 translation task compared to T1. This shift from production-oriented to reflection-oriented behavior suggests heightened metacognitive engagement, as students spent a greater proportion of task time pausing to plan, evaluate, and problem-solve (Sfeir et al., 2026). Notably, individual variation was observed. While the majority of participants showed an increased pause time proportion, three participants showed a slight decrease. However, despite these individual differences, the overall group trend was statistically significant, with a moderate effect size (Cohen's *d* = 0.61).

**Table 3:** Temporal effort measures from T1 to T2 (experimental group, *n* = 11).

Measure	T1 (*M*, SD)	T2 (*M*, SD)	MD (95%CI)	*t*(10)	*p*	Cohen's *d*
Pause time proportion	25.0% (12.0%)	33.4% (14.5%)	0.084 [0.024, 0.145]	3.09	0.011^*^	0.61

^*^p < 0.05.

#### 4.1.2 Technical effort

The third dimension of Krings' model, technical effort, was defined as the number and type of editing operations performed on the translated text (Krings, 2001; Mossop, 2014). Revision operations were analyzed from Inputlog revision logs and cross-validated with screen recordings. Following Mossop's (2014) classification, revisions were coded into four types: deletion, addition, movement, and adaptation. Additionally, following the distinction established in writing process research (Vandermeulen et al., 2023), revisions were classified as surface-level (affecting spelling, punctuation, or single-word substitutions) or meaning-level (involving syntactic restructuring, clause reordering, or semantic changes). An individual revision trajectory by this classification was reported based on the student's revision logs (Figure 2). The ratio of meaning-level revisions to total revisions was computed as an indicator of deep processing. A revision intensity heatmap was presented accordingly as part of visualization feedback (Figure 3). A paired-samples *t*-test was conducted to compare the ratio of meaning-level revisions and the total number of revisions between T1 and T2.

**Figure 2:**
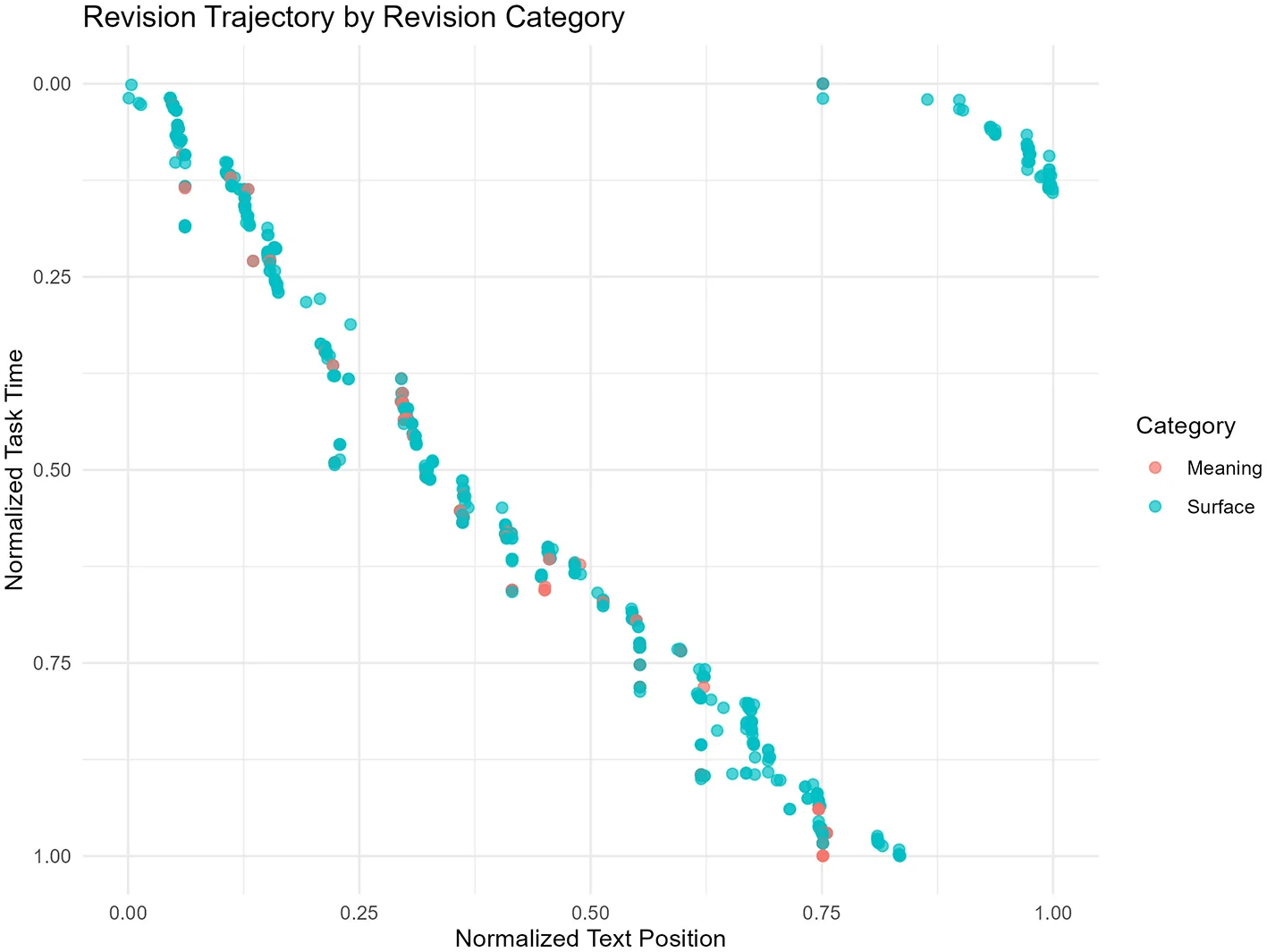
Changes in the proportion of external source consultation time by category. Experimental group (*n* = 11). Each box summarizes the group distribution; points are individual participants and dashed lines connect the same participant across the two time points.

**Figure 3:**
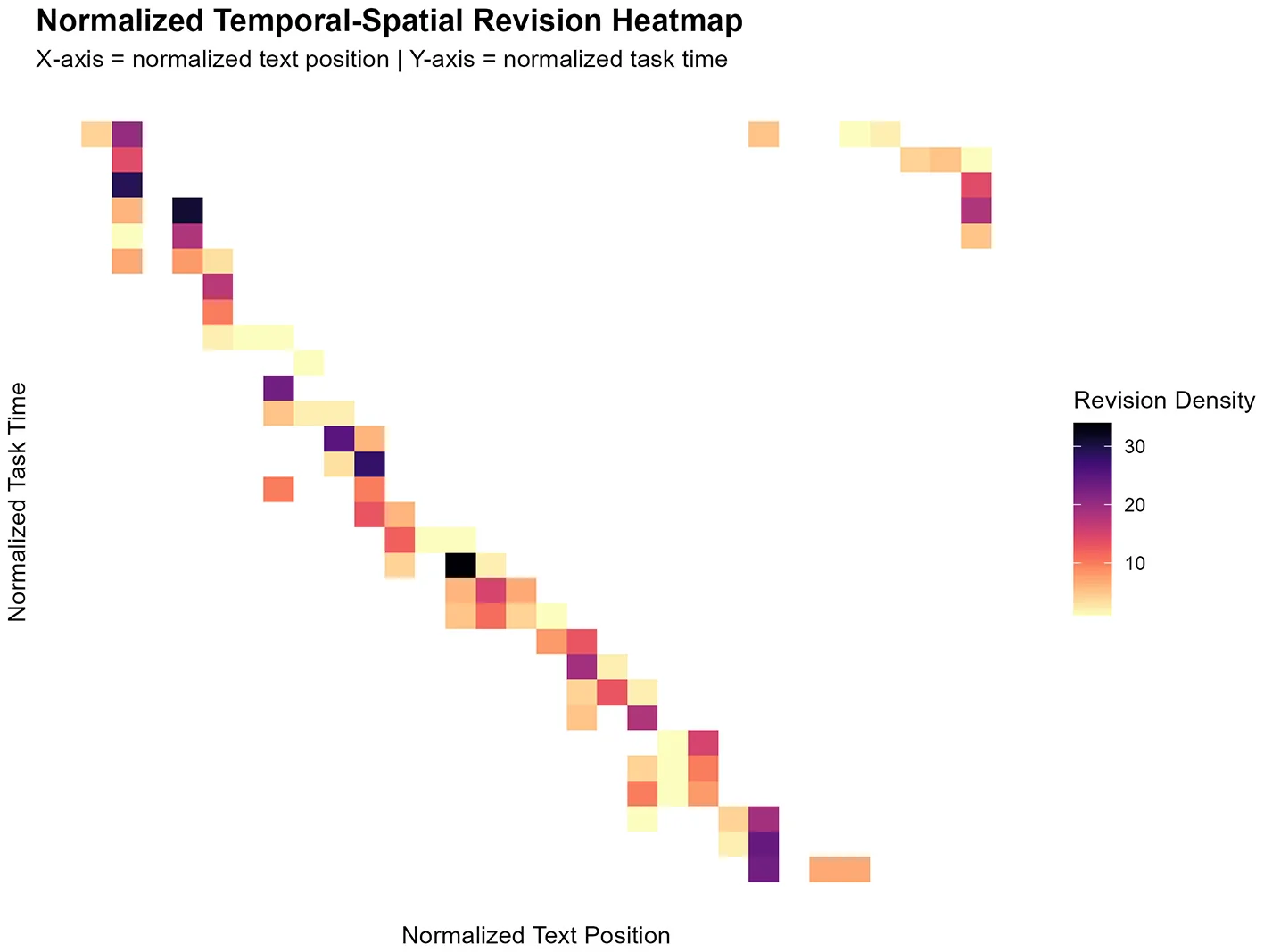
External source consultation visualization. Example of the visualization provided to students. The left panel shows the average proportion of task time invested in each consultation type; the right panel shows a transition network between consultation types, in which node size encodes total time spent and edge width encodes the number of switches between two types.

As shown in Table 4, the proportion of meaning-level revisions increased significantly from T1 (*M* = 18.1%, SD = 6.3%) to T2 (*M* = 28.4%, SD = 8.9%), with a mean increase of 10.31 percentage points [95% CI (4.92%, 15.7%)], *t* (10) = 4.259, *p* = 0.002, Cohen's *d* = 1.301, indicating a large effect. In contrast, the total number of revisions did not change significantly (T1: *M* = 1,292.91, SD = 394.24; T2: *M* = 1,052.36, SD = 331.91; *p* = 0.248, Cohen's *d* = −0.662). These results suggest that while the overall frequency of editing remained stable, the nature of revisions shifted toward deeper, meaning-oriented operations (e.g., syntactic restructuring, semantic changes) in the later stage of the translation task. These findings indicate that participants moved beyond superficial error correction toward deeper substantive textual refinement, a competency which is particularly important for news translation where editorial adaptation is central (Mossop, 2014; Vandermeulen et al., 2023).

**Table 4:** Revision patterns from T1 to T2 (experimental group, *n* = 11).

Measure	T1 (M, SD)	T2 (M, SD)	Mean deviation (95% CI)	*t*(10)	*p*	Cohen's *d*
Meaning ratio	18.1% (6.3%)	28.4% (8.9%)	0.103 [0.049,0.157]	4.259	0.002^**^	1.301
Total revisions	1,292.91 (394.24)	1,052.36 (331.91)	−240.55 [−677.07, 195.98]	−1.228	0.248	−0.662

^**^p < 0.01.

Although the proportion of meaning-level revisions increased significantly from T1 to T2, it still remained below that of surface-level revisions in both phases. Several factors may account for this persistent predominance of surface-level editing. First, the small sample size (*N* = 11) limits statistical power and the generalizability of the observed effect. With only 11 participants, individual variation could have masked a potentially larger shift toward deep-level revisions. Second, the 2-month time span between T1 and T2 might be insufficient for students to fully internalize and automatize deep-processing strategies, especially in a complex task such as news translation. A longer intervention period could be needed to observe a more balanced or even reversed pattern. Third, task-related demands, such as time pressure, text length, or assessment criteria may have encouraged students to prioritize quick, low-level corrections over laborious syntactic or semantic restructuring. Future studies with larger cohorts and extended longitudinal designs are warranted to determine whether the relative dominance of surface-level revisions is a stable phenomenon or one that can be overcome with more extended training and practice.

#### 4.1.3 Cognitive effort

The second dimension of Krings' model, cognitive effort, was operationalized through analysis of external source consultation behaviors. Following established procedures for analyzing information-seeking behaviors in translation (Ranalli et al., 2018), all external source consultation events were coded from Inputlog logs and screen recordings. A figure of student's external source consultation behavior (Figure 4) was generated based on student's information-seeking behaviors. By making normally invisible consultation patterns visible, the figure enabled students to identify strengths and weaknesses in their information-seeking behavior and conduct more strategic information seeking.

**Figure 4:**
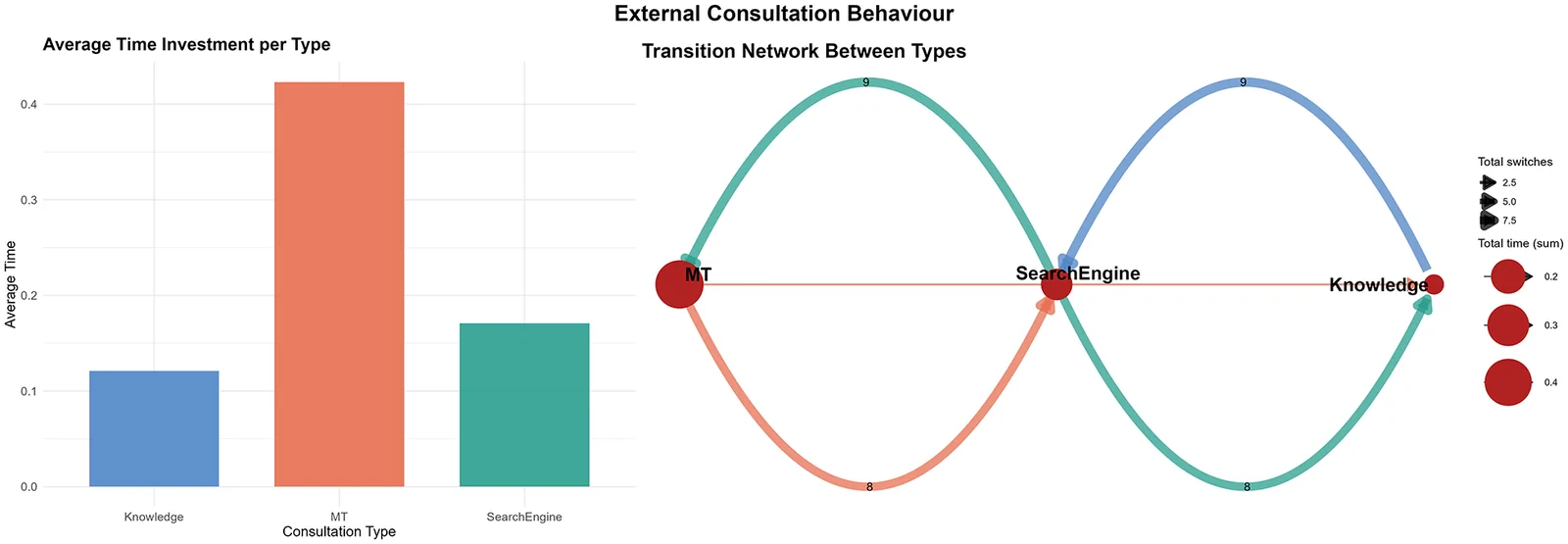
Changes in translation quality scores across time by group. Points are group means and error bars show 95% confidence intervals. The experimental group (*n* = 11) received process visualization feedback and the control group (*n* = 11) received conventional product-oriented feedback.

These external source consultation events were classified into five categories: machine translation (MT) use, search engine queries, knowledge-based searching, dictionary lookups, and interactive AI use. Interactive AI use was defined as instances where participants provided explicit prompts to large language models. In addition, the proportion of time before the first external consultation window was opened (“Before Window”) was included to capture participants' initial self-regulated processing prior to external resource consultation. For each participant, the proportional time allocated to each category was calculated for T1 and T2. Because the data consisted of small-sample proportional measures with substantial zero values, Wilcoxon signed-rank tests were conducted to compare T1 and T2 consultation patterns.

Table 5 shows substantial changes in participants' external consultation behaviors across the two time points. Compared with T1, participants devoted significantly more time to search engine queries in T2 (Median = 27.7%) than in T1 (Median = 15.6%), *V* = 3, *P*_holm_ = 0.020, *r* = 0.85. Knowledge-based searching also increased significantly from T1 (Median = 7.2%) to T2 (Median = 28.8%), *V* = 1, *P*_holm_ = 0.010, *r* = 0.93. In contrast, the proportion of time before opening the first external consultation window decreased significantly from T1 (Median = 12.1%) to T2 (Median = 1.7%), *V* = 60, *P*_holm_ = 0.041, *r* = 0.74, suggesting that participants initiated external consultation much earlier during the translation process at T2. MT use showed a noticeable decrease from T1 (Median = 28.5%) to T2 (Median = 5.1%), although the difference did not reach statistical significance after Holm correction, *V* = 41, *P*_holm_ = 0.338. Dictionary consultation remained relatively stable across the two time points, with no significant difference observed, *V* = 25, *P*_holm_ = 0.799. Interactive AI use remained limited overall, with the median proportion remaining at 0% in both T1 and T2, indicating that explicit prompting of large language models was concentrated among only a small subset of participants.

**Table 5:** Wilcoxon signed-rank tests of external source consultation categories.

Category	T1 Median	T2 Median	*V*	p_raw	p_holm	*Z*	*r*
MT	0.285	0.051	41	0.169	0.338	1.376	0.415
Search Engine	0.156	0.277	3	0.005	0.020^*^	2.815	0.849
Knowledge	0.072	0.288	1	0.002	0.010^*^	3.097	0.934
Before Window	0.121	0.017	60	0.014	0.041^*^	2.466	0.743
Dictionary	0.070	0.056	25	0.799	0.799	0.255	0.077

^*^p < 0.05 (Holm-corrected). Effect sizes were computed as *r* = *Z*/√*N*, where *N* = 11.

Taken together, these findings indicate a clear shift in participants' information-seeking strategies over time (Figure 5). Rather than relying primarily on direct lexical lookup resources such as MT and dictionaries, participants in T2 increasingly engaged in broader search engine consultation and knowledge-oriented information seeking. The simultaneous reduction in pre-consultation latency further suggests that participants developed a stronger tendency to initiate external cognitive support earlier in the translation process. Collectively, these changes point to the emergence of more strategic and cognitively distributed consultation behaviors over time.

**Figure 5:**
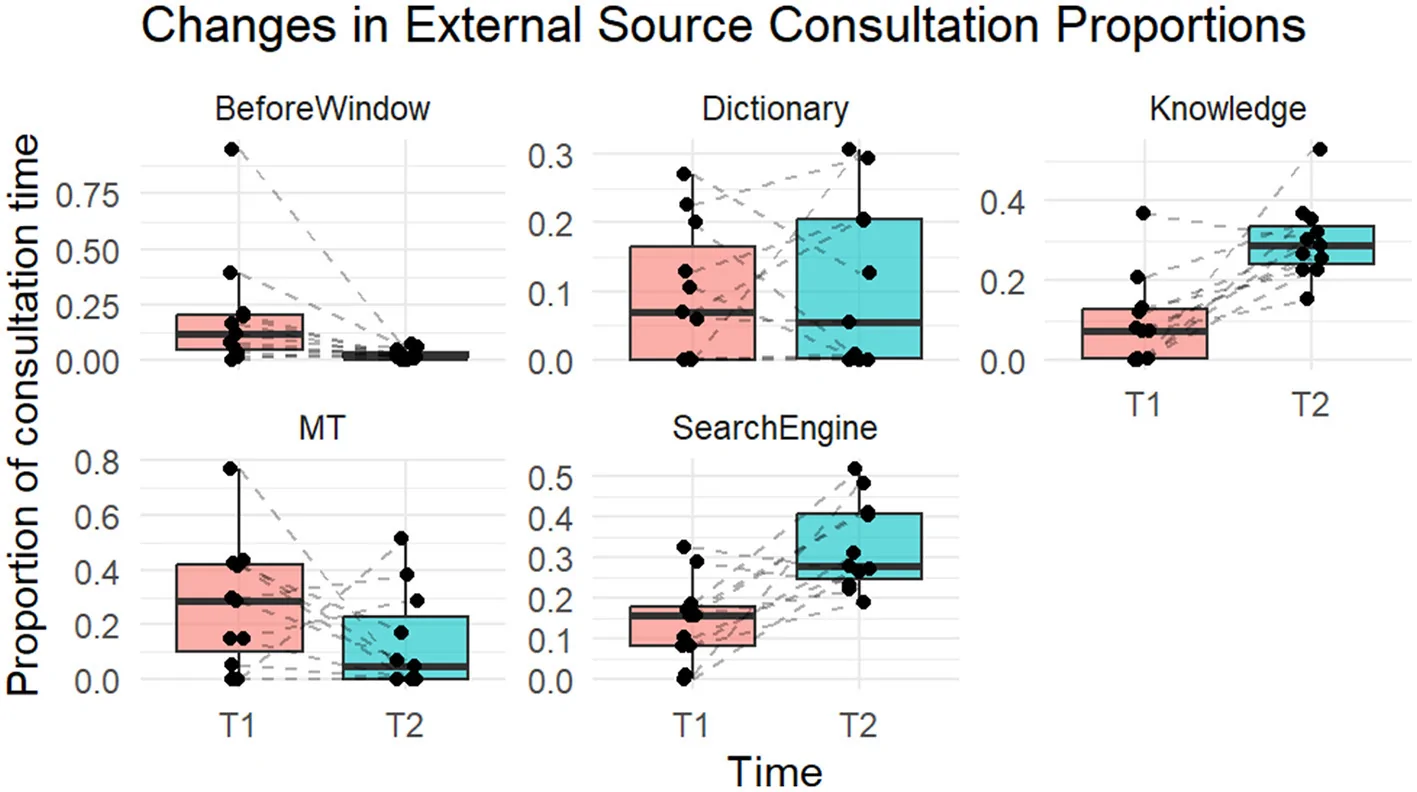
Individual revision trajectory by category. Shown for an example participant. Each point is one revision event, positioned by its location in the text (*x*-axis: normalized text position) and by when it occurred during the task (*y*-axis: normalized task time, reversed so that the task starts at the top). Pink points are meaning-level revisions and blue points are surface-level revisions.

A rich points analysis, following the PACTE translation competence model (Sfeir et al., 2026), was conducted through pause analysis (threshold set at ≥2,000 ms). To further examine cognitive effort during translation, pause episodes identified through Inputlog's pause analysis function were manually coded in combination with screen recordings according to the type of translation problem involved. Five categories of problems were identified: extralinguistic problems, linguistic problems, problems relating to the translation brief or target reader, textual problems, and intentionality problems. Linguistic problems concerned the accurate translation of terminology and proper nouns, whereas textual problems involved information structuring and discourse organization. Extralinguistic problems referred to culturally or politically sensitive issues requiring contextual interpretation and mediation. Intentionality problems concerned the alignment between authorial intent and translational purpose, while problems relating to the translation brief or target reader involved audience-oriented adaptation strategies. For each participant, the proportional distribution of pause-related problem types was calculated for T1 and T2. Wilcoxon signed-rank tests were conducted to compare differences across the two time points. Table 6 revealed several significant shifts in the distribution of problem-triggered pauses. Extralinguistic problems increased significantly from T1 (Median = 8.8%) to T2 (Median = 24.5%), *V* = 2, *P*_holm_ = 0.015, *r* = 0.90. Similarly, textual problems increased significantly from T1 (Median = 22.2%) to T2 (Median = 50.0%), *V* = 3, *P*_holm_ = 0.038, *r* = 0.75. In contrast, pauses related to the translation brief or target reader decreased significantly from T1 (Median = 20.8%) to T2 (Median = 2.6%), *V* = 63, *P*_holm_ = 0.020, *r* = 0.85. No significant differences were observed for linguistic problems or intentionality problems after Holm correction.

**Table 6:** Wilcoxon signed-rank tests of problem-triggered pauses.

Problem	T1 Median	T2 Median	*V*	p_raw	p_holm	*Z*	*r*
Extralinguistic problems	0.088	0.245	2	0.003	0.015^*^	2.975	0.897
Intentionality problems	0.000	0.014	8	0.161	0.323	1.400	0.422
Linguistic problems	0.385	0.189	46	0.278	0.323	1.084	0.327
Problems relating to translation brief or target reader	0.208	0.026	63	0.005	0.020^*^	2.815	0.849
Textual problems	0.222	0.500	3	0.013	0.038^*^	2.497	0.753

^*^p < 0.05 (Holm-corrected). Effect sizes were computed as *r* = *Z*/√*N*, where *N* = 11.

These findings (Figure 6) suggest that participants' cognitive effort gradually shifted from audience-oriented adaptation and local lexical concerns toward higher-level discourse organization and extralinguistic mediation, a shift which is particularly relevant for news translation where editorial adaptation is central. The increase in textual and extralinguistic problem-solving indicates growing engagement with information structuring, contextual interpretation, and ideological sensitivity, reflecting a more globally oriented approach to translation problem solving in T2. Moreover, the significant decrease of problems relating to translation brief or target reader reveals that students in experimental group in T2 become more familiar with the targets of the news trans-editing and the readers' expectation. Though the decrease of linguistic problems and increase of intentionality problems are not statistically significant, the development of these two problems could be identified as the trends, requiring experiment with larger sample to verify.

**Figure 6:**
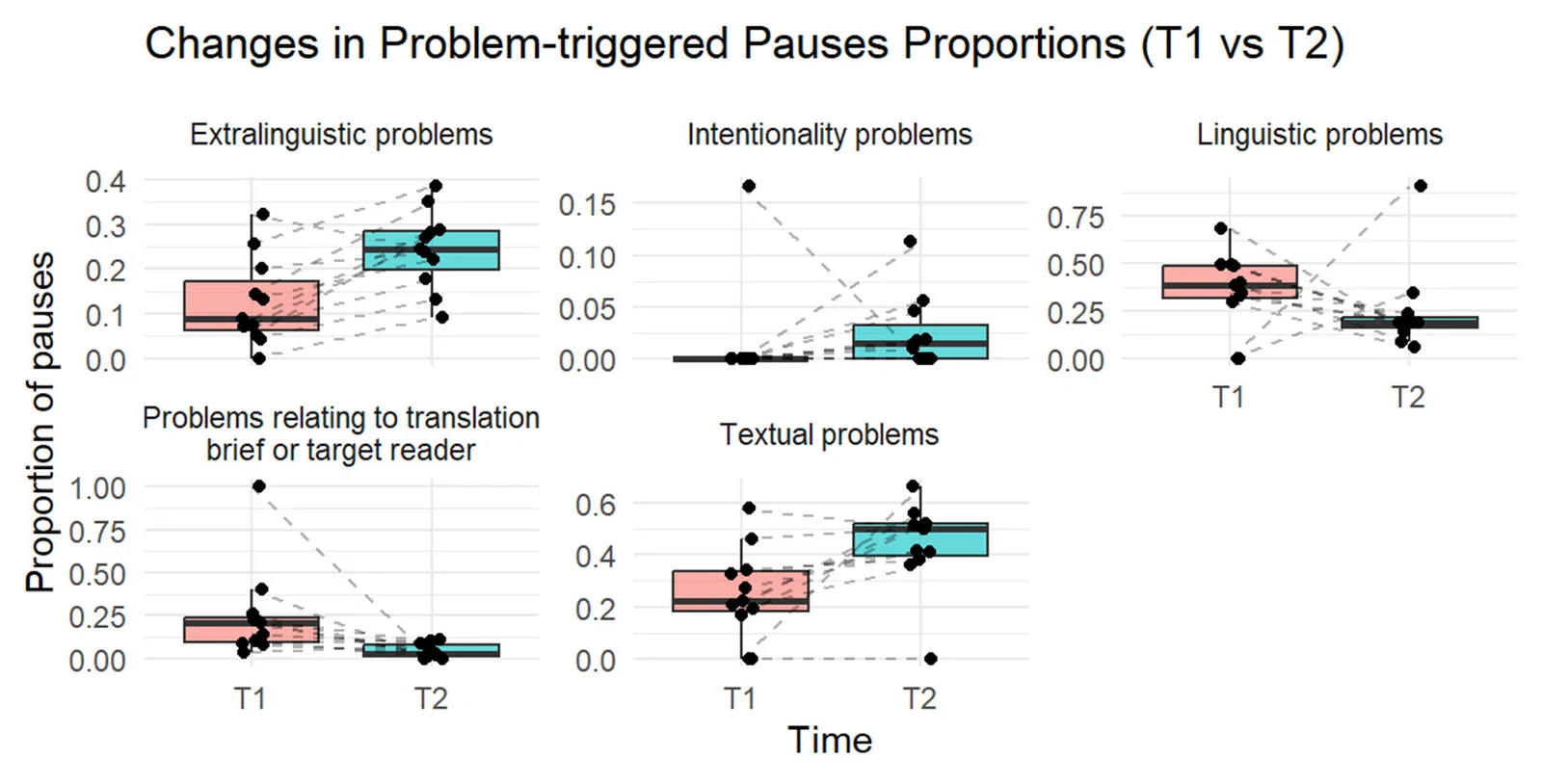
Normalized temporal-spatial revision heatmap. Shown for an example participant. The *x*-axis is the normalized text position and the *y*-axis is the normalized task time (reversed so that the task starts at the top). Color encodes the density of revision events, so darker cells mark where and when revisions clustered.

### 4.2 Analysis of outcome-level effects (RQ2)

To examine the impact of process visualization feedback on translation quality, a 2 (Group: experimental vs. control) × 2 (Time: T1 vs. T2) mixed design analysis of variance (ANOVA) was conducted. Translation quality score on a 0 to 100 scale served as the dependent variable.

Prior to conducting the mixed-design ANOVA, the assumptions of normality and homogeneity of variance were examined. Shapiro-Wilk tests indicated that translation scores in all four conditions were normally distributed, with no significant deviations from normality observed for the control group at T1 (*p* = 0.349) or T2 (*p* = 0.126), nor for the experimental group at T1 (*p* = 0.217) or T2 (*p* = 0.454). Homogeneity of variance was assessed using Levene's test. The results showed that the assumption of equal variances was satisfied for both T1, *F* (1, 20) = 0.17, *p* = 0.684, and T2, *F* (1, 20) = 0.20, *p* = 0.656. Because the within-subject factor (Time) contained only two levels (T1 and T2), the assumption of sphericity was automatically met and therefore did not require further testing. These results indicated that the data satisfied the assumptions required for mixed-design ANOVA.

The analysis (Table 7) revealed a significant main effect of Time, *F* (1, 20) = 20.24, *p* < 0.001, η*p*^2^ = 0.503, indicating that participants' translation scores generally improved from T1 to T2. Although the main effect of Group was not statistically significant, *F* (1, 20) = 3.54, p = 0.074, η*p*^2^ = 0.151, a significant Group × Time interaction was observed, *F* (1, 20) = 4.94, *p* = 0.038, η*p*^2^ = 0.198, suggesting that the two groups demonstrated different developmental trajectories over time. *Post-hoc* comparisons showed that the experimental group significantly improved from T1 to T2, *p* < 0.001, whereas the control group did not show a statistically significant improvement, *p* = 0.123. These findings indicate that process visualization feedback had a positive effect on the quality of students' translated news texts and facilitated greater improvement in translation performance.

**Table 7:** Mixed-design ANOVA results for translation quality scores.

Effect	df	*F*	*p*	Partial eta squared
Group	1, 20	3.54	0.074	0.151
Time	1, 20	20.24	< 0.001^***^	0.503
Group × Time	1, 20	4.94	0.038^*^	0.198

^*^p < 0.05. ^***^p < 0.001.

Moreover, the visualized interaction between Group and Time in translation quality was presented in Figure 7. Both groups demonstrated improvement from T1 to T2, but the experimental group exhibited a markedly steeper increase than the control group. This pattern is consistent with the significant Group × Time interaction observed in the mixed-design ANOVA, suggesting that process visualization feedback contributed to greater gains in translation performance over time.

**Figure 7:**
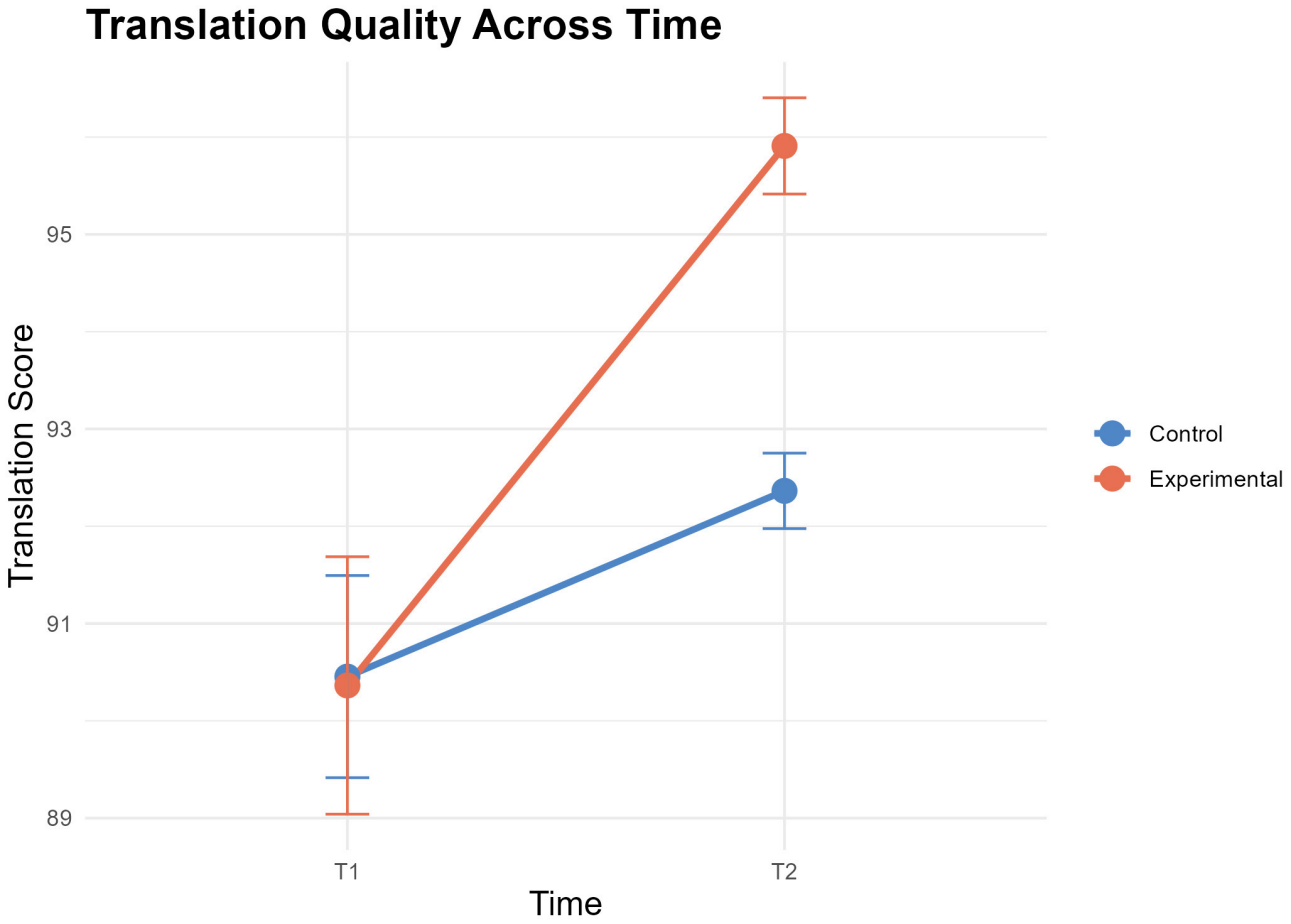
Changes in the proportion of problem-triggered pauses by problem type. Experimental group (*n* = 11). Each box summarizes the group distribution; points are individual participants and dashed lines connect the same participant across the two time points.

### 4.3 Analysis of perceptual and metacognitive effects (RQ3)

Reflective journals from the 11 experimental group participants, totaling 22 journals and approximately 15,000 words, and interview transcripts from 6 participants, totaling approximately 30,000 words, were analyzed using thematic analysis following the six-phase procedure established by Braun and Clarke (2006). Initial coding was conducted inductively using NVivo 15 qualitative data analysis software. A codebook was developed iteratively, with 20 percent of the data double-coded by a research assistant, yielding an inter-coder agreement κ of.86. Codes were then grouped into candidate themes, which were checked against coded extracts and the full dataset. Two overarching themes were identified: metacognitive awareness and strategic regulation. The coding scheme and theme structure are summarized in Table 8.

**Table 8:** Themes and subthemes from qualitative analysis.

Theme	Subtheme	Files	References
1.Metacognitive Awareness	1.1 Awareness of inefficient process patterns	12	14
	1.2 Awareness of specific competency gaps	18	40
2.Strategic Regulation	2.1 Strategic use of external sources	14	29
	2.2 Text-level restructuring and adaptation	12	22
	2.3 Changes to AI interaction patterns	6	9

Total files = 21 for metacognitive awareness (some files contained both subthemes) and 19 for strategic regulation.

The qualitative findings were interpreted through Winne and Hadwin's (1998) COPES model. Theme 1 captures awareness of operations and internal conditions, including evaluations against personal or task-related standards. Theme 2 captures reported strategic adjustments that can be interpreted as metacognitive control when linked to an identified difficulty, goal, or criterion. Following Nicol (2021), the analysis also considers how comparisons between recorded activity and participants' expectations or goals may have supported internal feedback generation.

#### 4.3.1 Theme 1: Metacognitive awareness

This theme concerns how participants used process visualizations to reconsider aspects of their translation activity that had previously received limited attention. Accounts varied from noticing repeated or uneven operations to evaluating their implications for performance, and some participants also recognized limitations in their knowledge or strategies. These forms of awareness provided potential starting points for regulation.

**Subtheme 1.1: Awareness of inefficient process patterns**. The most frequently reported form of metacognitive awareness was recognition of suboptimal process behaviors, especially in time allocation and resource use. Students described spending excessive time on unproductive activities such as switching repeatedly between tools, revising the same sentence without progress, or failing to plan before drafting. One participant remarked, “I did not realize how much time I wasted on back-and-forth dictionary lookups until I saw the visualization” (interview). This account combines retrospective monitoring with evaluation: the participant identified repeated consultation as a pattern and judged it unproductive. In COPES terms, it suggests monitoring of an operation and evaluation against an implied standard of efficient time use; comparing the visualization with prior understanding also illustrates how process evidence may support internal feedback generation (Nicol, 2021). Another participant wrote, “The report showed me that I spent almost 30 minutes on the first paragraph alone. I never noticed that during the task” (journal). Here, the report made the concentration of time on one passage available for retrospective monitoring and revealed an incomplete understanding of their own activity. These accounts are consistent with the quantitative increase in pause time proportion from T1 to T2 and suggest greater reflective attention to temporal management.

**Subtheme 1.2: Awareness of specific competency gaps**. Beyond process inefficiencies, students identified concrete knowledge and skill gaps. These included terminology (“I did not know the standard Chinese translation for ‘gigawatts”'), cultural knowledge (“I was unfamiliar with the background of the US-China balloon incident”), and strategic competence (“I realized I did not know how to verify AI-generated translations”). In COPES terms, these accounts concern internal conditions, including domain and strategic knowledge. Their metacognitive significance lies in participants' recognition that available knowledge did not meet task demands; such recognition may inform regulation but does not itself show that the gaps were addressed. A clearer link between process evidence and difficulty identification appeared in one interview: “Seeing my pauses on the timeline helped me identify exactly where I struggled. Those were the moments I did not know how to phrase a culturally sensitive term.” Such awareness was often linked to targeted strategy adjustments, reported in Theme 2.

#### 4.3.2 Theme 2: Strategic regulation

Whereas Theme 1 concerns identifying and evaluating difficulties, Theme 2 concerns reported adjustments to translation activity. Accounts were interpreted as strategic regulation when participants described a change in approach together with an identifiable purpose or criterion.

**Subtheme 2.1: Strategic use of external sources**. The most prominent reported change was more strategic and targeted use of external sources. Students described moving from single-source dictionary lookups to multi-source cross-verification, from passive reception to active retrieval of parallel texts, and from word-level to context-level information seeking. One participant stated, “In T2, I started searching for background information on green technology policies before translating, which helped me better understand the source text” (interview). Contextual information was thus sought before drafting to support understanding rather than to resolve an isolated lexical problem. Another wrote, “I used to just copy the first dictionary entry I saw. Now I check multiple sources and compare how terms are used in authentic Chinese news articles” (journal). This indicates a change in the basis for selecting expressions: cross-source comparison replaced immediate acceptance of the first result, and authentic news usage provided a reference for assessing possible translations. Regulation therefore involved a change in selection criteria, not merely increased resource use, and illustrates Nicol's (2021) argument that reference information can support internal feedback through comparison with one's own developing work. These accounts align with the quantitative increases in search engine and knowledge-based searches.

**Subtheme 2.2: Text-level restructuring and adaptation**. Participants also reported more frequent text-level restructuring, moving beyond sentence-by-sentence translation toward discourse-level reorganization. They described condensing redundant information, reordering sentences to improve logical flow, and adapting expressions for target readers—skills central to news translation (Bielsa and Bassnett, 2011). One participant wrote, “I no longer translate sentence by sentence. Instead, I read through the whole text first, understand the logical relationships, and then reorganize the Chinese expression” (journal). This adjustment concerns the planning and coordination of translation activity rather than the correction of individual sentences. Another explained, “For the green tech article, I combined several short paragraphs into one because that is how Chinese news is written” (interview). In COPES terms, this connects an operation (combining paragraphs) to a participant-articulated standard for the target text. These accounts illuminate reported reasoning behind some restructuring decisions, while the revision measures document the broader within-group change in revision types.

**Subtheme 2.3: Changes to AI interaction patterns**. Evidence of change in AI interaction was more limited. A small number of students reported deliberate changes in their interaction with AI tools, suggesting emerging but not yet widespread development of AI collaboration strategies following process visualization feedback. One participant noted, “I started trying to use more specific prompts with ChatGPT instead of just pasting the text and asking for a translation. I asked it to explain why it made certain choices” (interview). In COPES terms, this suggests a deliberate modification of the prompting operation with the stated purpose of obtaining explanations for AI-generated choices. However, it does not establish how the participant evaluated those explanations or whether they improved subsequent decisions. Together with the limited quantitative evidence, this supports an interpretation of participant-specific adjustments rather than group-wide development of AI collaboration competence.

Across the accounts, a possible metacognitive sequence emerges: reviewing process evidence made previously unnoticed activity available for examination; participants identified inefficiencies or knowledge gaps, sometimes evaluated them against task-related standards, and reported adjustments in subsequent practice. In COPES terms, these accounts connect monitoring and evaluation with potential metacognitive control.

#### 4.3.3 Triangulation of qualitative and quantitative findings

To triangulate the qualitative findings with process data, critical incidents identified in the reflective journals were cross-referenced with Inputlog logs and screen recordings. For example, when a participant reported in their T2 journal that they had started searching for background information before translating, the screen recording was reviewed to verify this behavioral change, and the Inputlog logs were examined to measure its impact on pause patterns and revision quality. This triangulation procedure enhanced the validity of the qualitative interpretations by grounding self-reported changes in observable behavioral data.

## 5 Discussion

This study investigated the effects of process visualization feedback on human-AI interaction in news translation tasks, framed within Krings' (2001) three-dimensional model of post-editing effort. Within the intervention group, the findings revealed significant changes across all three dimensions: temporal (increased pause time proportion), technical (increased meaning-level revision ratio), and cognitive (more strategic use of external sources). These results suggest that process visualization feedback was associated with enhanced metacognitive awareness and strategic regulation in human-AI collaborative translation. Below, each dimension is discussed in relation to existing literature, followed by an examination of integrated effects across dimensions, theoretical contributions, and pedagogical implications.

### 5.1 Enhanced metacognitive engagement in the temporal dimension

One of the most striking findings of this study was the significant shift in students' temporal effort from T1 to T2 with pause time proportion increasing from 25.0% to 33.4% (*p* = 0.011). At first glance, this might appear to indicate reduced efficiency. However, this finding is interpreted as evidence of heightened metacognitive engagement rather than inefficiency.

This interpretation is consistent with previous research on expert-novice differences in translation. Expert translators tend to spend proportionally more time on orientation and end revision, whereas novices often devote excessive time to drafting due to inadequate planning (Jakobsen, 2002; Carl et al., 2016). This shift is consistent with students moving from a production-oriented approach toward a more reflective approach, characterized by increased planning, evaluation, and problem-solving during pauses. As Sfeir et al. (2026) argued, increased pause time may indicate heightened metacognitive engagement rather than inefficiency, as translators pause to plan, evaluate, and problem solve.

The qualitative data provided converging evidence for this interpretation. In their reflective journals and interviews, students explicitly reported becoming aware of inefficient time allocation patterns. One participant noted, “I did not realize how much time I wasted on back-and-forth dictionary lookups until I saw the visualization.” Another wrote, “The report showed me that I spent almost 30 min on the first paragraph alone. I never noticed that during the task.” These self-reports, triangulated with Inputlog data, support the interpretation that the increased pause time proportion reflected strategic reflection rather than confusion or task difficulty.

This finding extends previous research on process feedback in writing. Vandermeulen et al. (2023) found that providing students with personalized process reports yielded meaningful improvements in text quality. Speltz and Chukharev-Hudilainen (2021) reported that real-time fluency-focused feedback helped students produce more text and higher-quality texts. This study extends these findings to translation education, by showing that process visualization feedback was accompanied by a shift in students' temporal allocation patterns toward more reflective engagement. Importantly, this shift did not compromise task completion, as all participants finished the translation within the allocated time, suggesting that increased reflection can be integrated productively within existing time constraints.

### 5.2 Deepened processing in the technical dimension

The second major finding concerns the nature of revision behavior. While the total number of revisions remained stable from T1 to T2, the ratio of meaning-level revisions to total revisions increased significantly from 18.1% to 28.4% (*p* = 0.002, Cohen's *d* = 1.301). This indicates that participants moved beyond surface-level error correction toward deeper substantive textual refinement. The effect size is particularly noteworthy, indicating a substantial change in students' revision strategies following the intervention.

This finding is especially significant for news translation, where editorial adaptation is central (Bielsa and Bassnett, 2011; Schäffner, 2012). Unlike other translation genres that may prioritize fidelity to the source text, news translation requires practitioners to reorganize, condense, and rewrite content for target audiences. The ability to move beyond surface-level error correction toward deeper structural and stylistic revisions distinguishes more effective from less effective news translators (Mossop, 2014; Vandermeulen et al., 2023). The observed increase in meaning-level revisions suggests that students became more likely to recognize opportunities for substantive textual refinement that they had previously overlooked.

The qualitative data further illuminated this shift. Students reported moving beyond sentence-by-sentence translation toward discourse-level reorganization. One participant wrote, “I no longer translate sentence by sentence. Instead, I read through the whole text first, understand the logical relationships, and then reorganize the Chinese expression.” Another explained, “For the green tech article, I combined several short paragraphs into one because that is how Chinese news is written.” These accounts suggest that process visualization feedback helped students recognize the need for text-level restructuring and equipped them with strategies to achieve it.

Notably, the stability of total revision counts despite the qualitative shift in revision types suggests that students did not simply revise more; rather, they revised differently. This distinction is crucial, as it indicates a strategic reallocation of revision effort rather than a mere increase in revision quantity. Such strategic differentiation is a hallmark of developing expertise in translation (Mossop, 2014).

### 5.3 Strategic regulation in the cognitive dimension

The third major finding concerns cognitive effort operationalized through source consultation behaviors. Participants made significantly more search engine queries (*p* = 0.02) and knowledge-based searches (*p* = 0.01) in T2 compared to T1, while dictionary lookups decreased. This pattern indicates a shift from dictionary-dependent lookup behavior toward more strategic use of diverse information sources.

This finding resonates with Ranalli et al. (2018), who found that keystroke logging combined with eye-tracking facilitated learner development. The concept of rich points, which refers to source-text elements containing translation problems that reveal underlying decision-making (PACTE Group, 2005; Sfeir et al., 2026) captures how translators navigate linguistic, textual, and extralinguistic challenges. Following Enbaeva's (2021) classification of rich points into linguistic, extralinguistic, textual, intentionality, and translation brief problems, the analysis of problem-triggered pauses (Table 6) revealed a significant shift from T1 to T2: extralinguistic problems increased from 8.8% to 24.5% (p =0.015) and textual problems from 22.2% to 50.0% (*p* = 0.038), while translation-brief problems decreased (*p* = 0.020). This is consistent with a shift in students' attention from word-level and audience-adaptation concerns toward discourse-level and cultural challenges, a shift particularly valuable for news translation, where cultural mediation is paramount.

The qualitative data strongly supported this interpretation. The subtheme “strategic use of external sources” emerged as the most prominent strategic change reported by participants (14 files, 29 references). Students described moving from single-source dictionary lookups to multi-source cross-verification, from passive reception to active retrieval of parallel texts, and from word-level to context-level information seeking. One participant stated, “In T2, I started searching for background information on green technology policies before translating, which helped me better understand the source text.” Another wrote, “I used to just copy the first dictionary entry I saw. Now I check multiple sources and compare how terms are used in authentic Chinese news articles.”

A noteworthy finding emerged regarding interactive AI use. While its frequency increased from T1 to T2, this change was not statistically significant, and overall frequency remained low. This suggests that while process visualization feedback raised awareness of AI tools as potential resources, students may not have developed sufficient proficiency in identifying and correcting MT errors, a challenge also noted in previous research (Mellinger, 2017; Yang et al., 2023), or the prompting skills needed to engage effectively with LLMs (Abdelhalim et al., 2025). Moreover, students' continued reliance on search engines and knowledge bases may reflect a preference for traceable sources when verifying factual information and comparing translation options. This shift toward higher-order problem types aligns with human-centered AI approaches that position translators as strategic agents rather than passive recipients of machine output (Briva-Iglesias and O'Brien, 2024; Jiménez-Crespo, 2025). However, the present data cannot determine whether students' choices reflected concerns about AI hallucinations, prompting difficulties, or both.

### 5.4 Integrated effects across dimensions and translation quality gains

The three dimensions of effort examined in this study are not independent but mutually reinforcing. The shift toward more reflective temporal engagement (increased pause time proportion) likely enabled deeper cognitive processing (more strategic source consultation) and more substantive technical revision (increased meaning-level revisions). Conversely, more strategic source consultation may have required additional pause time, while deeper revision work may have prompted further resource consultation. This interpretation is consistent with the view of translation as a complex cognitive activity in which temporal, technical, and cognitive dimensions are dynamically interrelated (Krings, 2001).

The triangulation of quantitative and qualitative data provides strong support for this integrated interpretation. Students who reported greater metacognitive awareness of their process patterns also demonstrated larger gains in meaning-level revision ratio and more strategic source consultation. The convergence of evidence from different data sources therefore strengthens the interpretation of these within-group process changes. The causal claim about the intervention's effect on translation quality rests on the between-group comparison reported in Section 4.2, in which both groups were assessed at both time points.

A significant Group × Time interaction was observed (*p* = 0.038, η*p*^2^ = 0.198) through a mixed-design ANOVA, which provides outcome-level validation that these process-level changes translated into tangible product improvements. Notably, both groups improved from T1 to T2, which is expected given task repetition and general learning effects. However, the experimental group improved significantly (*p* < 0.001), with a mean gain of 5.64 points compared to 1.91 points for the control group. This indicates that the intervention had a practically meaningful impact on translation quality beyond the natural improvement observed in the control group. The gain suggests improved editorial readiness within an already high-scoring sample. However, as the group mean remained within the same broad readiness band and editing time was not measured, the findings cannot quantify a reduction in newsroom editing workload.

The significant Group × Time interaction effect and the significant difference in gain scores provide more sensitive tests of the intervention's impact, as they account for baseline differences. Taken together, the results provide evidence of greater improvement in translation quality under the process visualization and structured reflection condition than under conventional product-oriented feedback.

### 5.5 Theoretical contributions

This study makes several theoretical contributions. The first contribution extends the application of Krings' (2001) three-dimensional effort model from post-editing research to the design and evaluation of pedagogical interventions. By operationalizing temporal, technical, and cognitive effort as measurable indicators of feedback effectiveness, this study demonstrates how process data can be used not only to describe translation processes but also to assess instructional interventions. This methodological innovation opens new avenues for process-oriented translation pedagogy research.

The study also contributes to the growing body of research on process-oriented feedback in translation education (Feng, 2016; Lindgren et al., 2009). While previous studies have demonstrated the potential of keystroke logging and screen recording for raising student awareness, controlled designs that allow the effects of such feedback to be isolated are still relatively uncommon. The pretest-posttest control group design employed here addresses this gap at the level of the translated product, providing empirical support for an intervention effect on translation quality, while the process and qualitative data trace how metacognitive awareness and strategic regulation developed within the intervention group. The inclusion of qualitative data further strengthens this interpretation by revealing the mechanisms through which these enhancements occurred.

A further contribution advances understanding of human-AI collaboration in translation. Grounded in a human-centered AI approach that positions translators as strategic agents rather than passive recipients of machine output (Briva-Iglesias and O'Brien, 2024; Jiménez-Crespo, 2025), this study demonstrates that process visualization feedback can help students develop this competence by making visible their interactions with AI tools and other resources. The observed shift in students' cognitive effort, from lower-order linguistic concerns to higher-order textual and extralinguistic problems, exemplifies this development, as learners learned to engage with the kinds of context-dependent challenges that remain beyond the reach of current AI. The increased strategic use of external sources, including AI tools, further indicates that students positioned themselves as active agents in the translation process rather than passive recipients of machine-generated output.

### 5.6 Pedagogical implications

The findings of this study have several implications for translator education. Process visualization feedback can be integrated into existing translation curricula as a formative assessment tool. The personalized process reports used in this study required a relatively modest time investment, a 30-min individual session per student, but was associated with changes in all three dimensions of effort and with higher translation quality. This suggests that process visualization feedback is feasible and potentially effective in educational settings, even within the constraints of existing course structures.

The structured reflection log that accompanied the process visualization report appeared to play a crucial role in helping students translate observations into action plans. Rather than simply showing students their process data, the intervention guided them to identify specific areas for improvement and formulate concrete goals for the subsequent task. This aligns with the principle that effective feedback must empower learners to monitor and regulate their own learning (Black and Wiliam, 1998; Nicol and Macfarlane-Dick, 2006). Without such structured guidance, process data alone may overwhelm or confuse students, potentially leading to superficial interpretations.

The sequence through which the intervention operated can be summarized as translation, feedback, revision, and reflection (Figure 8). Students first completed a news translation task whose process was recorded; they then received a personalized visualization of that process together with a structured reflection log, which they used to identify specific weaknesses and to formulate action plans; they applied those plans while revising their own practice in subsequent course assignments; and they finally reflected on what had changed at the second measurement point. Making this sequence explicit matters because it locates the point at which feedback becomes useful: the visualization on its own is only information, and it is the reflection that follows it, together with the opportunity to act on it during revision, that turns that information into a change in behavior.

**Figure 8:**
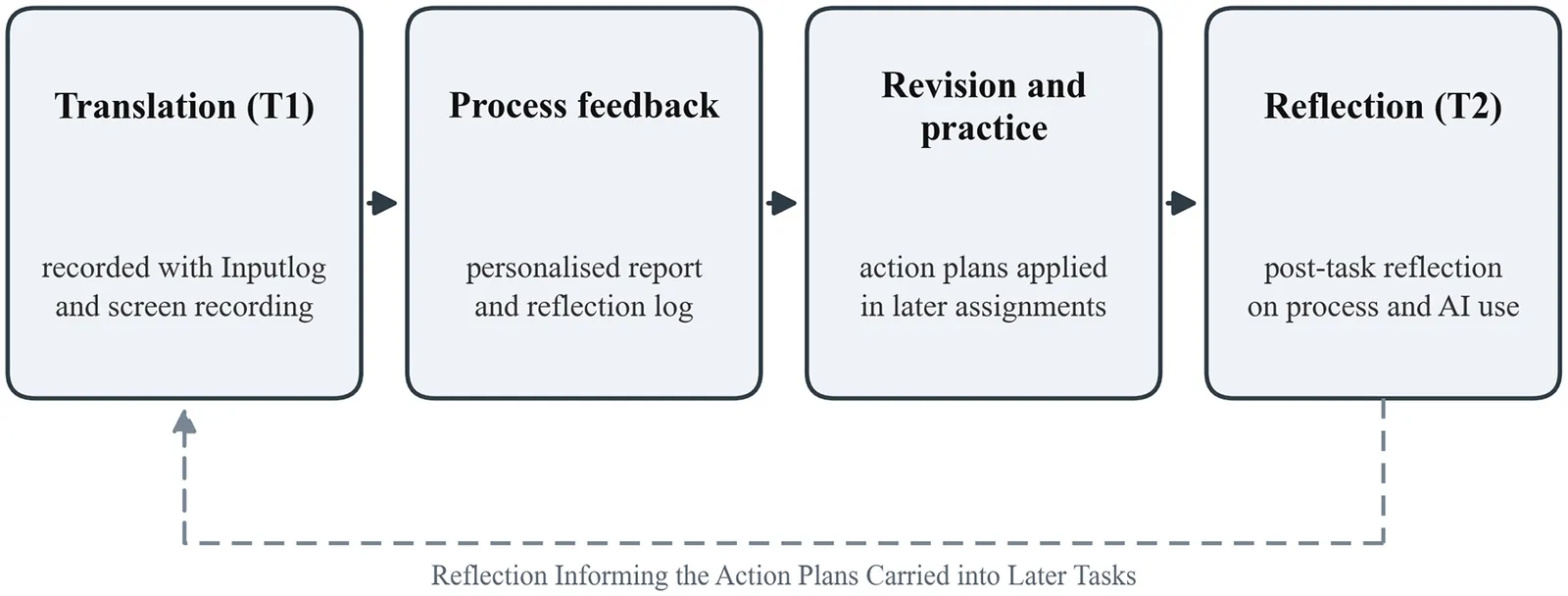
The pedagogical sequence of the intervention: translation, feedback, revision, and reflection. The dashed arrow indicates that reflection informs the action plans carried into subsequent translation tasks.

The findings also highlight the importance of explicitly teaching resource consultation strategies. While students naturally increased their use of search engines and knowledge-based resources following the intervention, many struggled to use AI tools effectively. The low frequency of interactive AI use and the small number of students reporting changes in AI interaction patterns suggest that developing AI collaboration competence requires more than process awareness alone. Translator education programs should devote explicit attention to developing students' AI literacy, including how to formulate effective prompts, evaluate AI-generated output critically, and integrate AI tools strategically into the translation workflow.

Finally, the differential patterns of improvement observed across the three dimensions of effort suggest that process visualization feedback may have cascading effects. The temporal dimension showed the most immediate change, followed by the cognitive and technical dimensions. This pattern may reflect the nature of the intervention: time allocation patterns may be more easily modified than deeply ingrained revision habits or information-seeking strategies. Educators should be aware that process-level changes may unfold over different time courses and adjust their expectations and instructional support accordingly.

### 5.7 Limitations and future directions

Several limitations of this study should be acknowledged. The most important limitation concerns the process-data design. Process data were collected from both groups at T1 but only from the experimental group at T2. The RQ1 findings therefore describe within-subject change in the intervention group and cannot be compared with a control baseline. Consequently, these findings alone cannot rule out task repetition, increasing familiarity with the news genre, or maturation as alternative explanations for the observed shifts in pausing and revision behavior. Only the product-level comparison, for which both groups were assessed at both time points, supports a causal claim about the intervention. Future studies should log control participants at both time points so that the specific effects of process visualization feedback can be separated from task-repetition effects on pause and revision distributions. One limitation concerns the sample size, which, while sufficient for detecting the observed effect sizes, was relatively small and drawn from a single institutional context with MTI students in China. Replication studies with larger and more diverse samples across different educational settings would enhance generalizability. Another limitation pertains to the intervention period, as the eight-week duration, while adequate for detecting short-term effects, does not address the durability of the observed changes. Longitudinal studies are needed to examine whether improvements are sustained over time and whether further gains occur with continued practice. An additional limitation concerns the scope of outcome measures, as the study focused on individual translation performance. Future research could examine whether process visualization feedback also enhances collaborative translation processes or influences how students provide feedback to peers. What's more, participants' baseline translation scores were high, averaging approximately 90 out of 100 in both groups. This restricted the available room for improvement and raises the possibility of ceiling effects. Future studies should include learners with a broader range of initial proficiency.

A noteworthy finding that also points to a limitation is the persistently low level of interactive AI use despite the intervention, suggesting that developing AI collaboration competence is a complex endeavor that may require more targeted instructional support beyond general process visualization feedback. Future research could investigate what types of AI-specific instruction are most effective for enhancing students' AI literacy, including how to formulate effective prompts, evaluate AI-generated output critically, and integrate AI tools strategically into the translation workflow. Design-based research approaches could be particularly valuable for developing and refining pedagogical interventions that target AI collaboration skills.

## 6 Conclusion

This study examined the effects of process visualization feedback on human-AI interaction in news translation tasks, framed within Krings' (2001) three-dimensional model of post-editing effort. A pretest–posttest control group design was employed with 24 MTI students; the final analysis included 22 participants (11 per group). Both groups had complete T1 and T2 translation scores, while the experimental group also had complete T1 and T2 process data.

Within the intervention group, significant changes were observed across all three dimensions of Krings' model. Temporally, students shifted from production-oriented to reflection-oriented behavior, as reflected in an increased pause time proportion (25.0% to 33.4%, *p* = 0.011), a pattern consistent with heightened metacognitive engagement. Technically, the meaning-level revision ratio increased substantially from 18.1% to 28.4% (*p* = 0.002, Cohen's *d* = 1.301), indicating a transition from surface-level error correction to deeper substantive textual refinement. Cognitively, students made more strategic use of external sources, with significant increases in search engine queries (*p* = 0.02) and knowledge-based searches (*p* = 0.01), a pattern corroborated by qualitative references (14 files, 29 references). In addition, analysis of problem-triggered pauses revealed a significant shift from linguistic and audience-related concerns toward textual and extra-linguistic problems (*p* = 0.038, *p* = 0.015), consistent with enhanced strategic regulation. These process-level changes translated into meaningful product-level outcomes. These process-level changes coincided with meaningful product-level improvement. The significant Group × Time interaction effect (*p* = 0.038, η*p*^2^ = 0.198) and the significant gain in the experimental group (*p* < 0.001) provided evidence of greater improvement in translation quality under the intervention than under conventional product-oriented feedback.

Thematic analysis of the qualitative data indicated that process visualization feedback was associated with metacognitive awareness, as students reported recognizing inefficient process patterns and specific competency gaps, and with strategic regulation, including more strategic use of external sources, text-level restructuring, and emerging changes to AI interaction patterns. Triangulation of quantitative and qualitative data provided converging evidence for the intervention's association with these changes.

Taken together, these findings suggest that process visualization feedback was associated with enhanced metacognitive awareness and strategic regulation across the temporal, technical, and cognitive dimensions of human-AI collaborative translation. By rendering invisible translation processes visible, analyzable, and actionable, this approach may support students in becoming more reflective, strategic, and autonomous learners. The study underscores the pedagogical value of process-oriented, data-driven feedback in translator education, particularly for developing effective human-AI interaction strategies in the complex and time-sensitive domain of news translation.

## Data Availability

The datasets analyzed in this study are not publicly available because the screen recordings, keystroke logs, and interview transcripts contain potentially identifiable information. De-identified summary data and excerpts supporting the findings may be obtained from the corresponding author upon reasonable request, subject to institutional ethical approval.
